# Giant Posterior Inferior Cerebellar Artery Aneurysm Mimicking a Brainstem Tumor

**DOI:** 10.7759/cureus.22706

**Published:** 2022-02-28

**Authors:** Marcos V Sangrador-Deitos, Luis A Rodríguez Hernández, Juan C Balcázar-Padrón, Armando Ruiz-Treviño, Edgar Nathal

**Affiliations:** 1 Vascular Neurosurgery, Instituto Nacional de Neurología y Neurocirugía "Manuel Velasco Suárez", Mexico City, MEX

**Keywords:** compression, brainstem, thrombosed, pica, giant intracranial aneurysm

## Abstract

Aneurysms from the vertebrobasilar system are rare, accounting for only 5%-10% of all intracranial aneurysms. The most common sites in which these lesions occur are the bifurcation of the basilar artery and the origin of the posterior inferior cerebellar artery (PICA). When the aneurysms present in the distal portion of the PICA, they represent from 0.5% to 6%. These aneurysms are called giant when they exceed 25 mm in diameter. We present a case of a 49-year-old male who presented with acute obstructive hydrocephalus, which required ventriculoperitoneal shunting and left hemispheric cerebellar syndrome. The magnetic resonance imaging study revealed an occupative mass located in the fourth ventricle, and diagnostic angiography showed a partially thrombosed giant saccular aneurysm in the posterior inferior cerebellar artery. He underwent surgical management via a lateral suboccipital approach. The aneurysm was remodeled and clipped successfully without complications, with an uneventful postoperative course.

Although rare, PICA aneurysms should always be considered when posterior fossa syndrome occurs, including brainstem and cranial nerve compression symptoms. It can easily be misdiagnosed as a neoplastic lesion, especially when the aneurysm reaches big or giant size. Therefore, complete diagnostic studies, such as cerebral angiography, must be performed. Surgical clipping must be offered as the first line of treatment. It provides occlusion of the aneurysm and relieves compressive symptoms.

## Introduction

The posterior inferior cerebellar artery (PICA) is the largest branch of the vertebral artery and is divided into five segments: anterior medullary, lateral medullary, tonsillomedullary, telovelotonsillar, and cortical [1]. PICA aneurysms are rare, accounting for only 0.5%-3% of all intracranial aneurysms, with those arising in the distal segment comprising 0.5%-6% for all PICA aneurysms [2]. While most intracranial aneurysms arise from arterial bifurcations, distal PICA lesions are most frequently seen in the telovelotonsillar segment, which comprises the tonsil's medial surface to the fissures between vermis, tonsil, and hemisphere, where it reaches the suboccipital surface. This happens due to the increased shear stress observed in the cranial loop that the course of this artery presents [3].

## Case presentation

**Figure 1:**
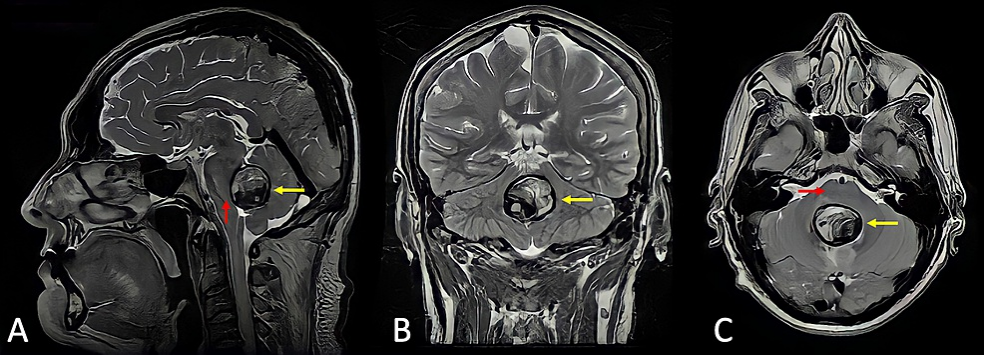
Magnetic resonance imaging (A) Sagittal, (B) coronal, and (C) axial T2 weighted sequence, which shows a 27-mm solid, heterogeneous, and occipital lesion fully occupying the fourth ventricle (yellow arrows). Brainstem displacement and edema can be observed (red arrows).

A 49-year-old man was admitted to our emergency department with an intense, new-onset headache, followed by nausea, vomiting, and altered consciousness. A CT scan revealed acute hydrocephalus due to an occupative lesion that filled the fourth ventricle. Emergency ventriculoperitoneal shunting was performed with no further complications. Postoperatively, the presenting symptoms improved, but the patient remained with left dysmetria, dysdiadochokinesia, and lateropulsion. A left hemispheric cerebellar syndrome was established. MRI revealed a heterogeneous tumoral lesion, wholly occupying the fourth ventricle, with brainstem compression and edema (Figure 1A-C).

**Figure 2:**
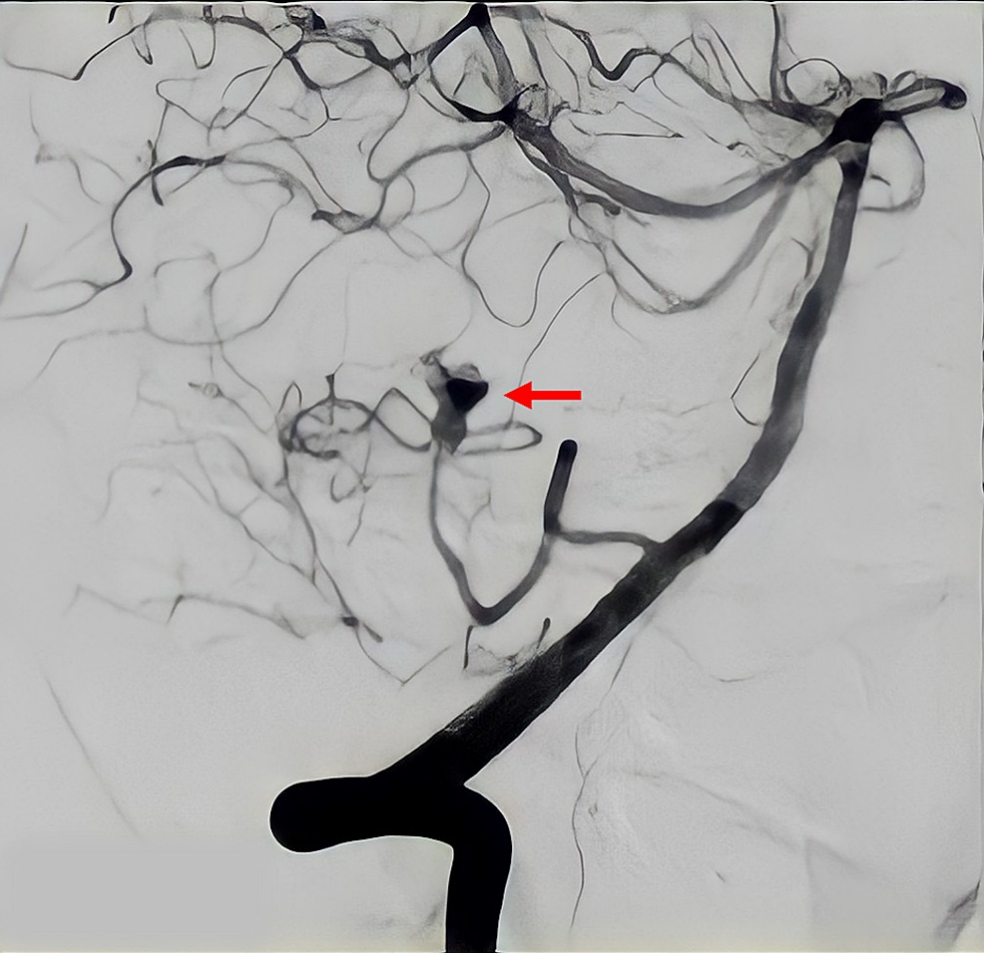
Digital subtraction angiography Left vertebrobasilar axis. A remnant neck of a partially thrombosed aneurysm in the telovelotonsillar segment of the PICA is observed (red arrow).

Diagnostic cerebral angiography revealed a 27-mm giant, partially thrombosed, saccular aneurysm in the left PICA (Figure 2).

**Figure 3:**
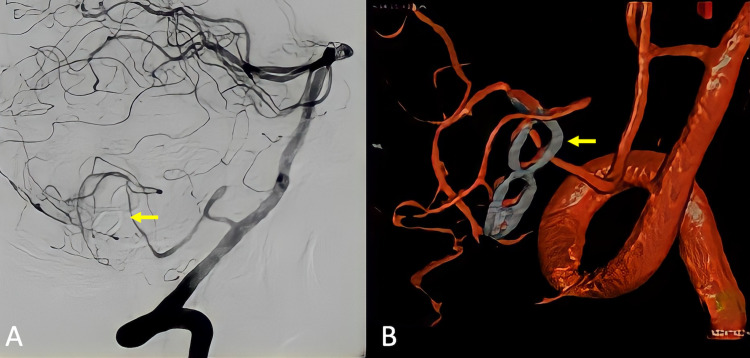
Postoperative imaging studies (A) Digital subtraction angiography and (B) CT angiography with 3D reconstruction showing complete aneurysm exclusion from the vertebrobasilar circulation and distal vessel's patency is confirmed. The 7-mm fenestrated clip can be observed occluding the aneurysm's neck (yellow arrows).

The patient was operated on via a lateral suboccipital approach, in which the angiographic findings were corroborated: a giant saccular aneurysm in the fourth segment of the PICA. Surgical clipping of the remnant neck with a 7-mm fenestrated clip was uneventful, and remodeling the aneurysm's dome relieved the mass effect exerted over the brainstem. Postoperative CT angiography revealed aneurysm exclusion and patency of distal vessels (Figure 3).

The cerebellar symptoms improved in the following weeks after the operation.

## Discussion

Aneurysms from the vertebrobasilar system are rare, accounting for only 5%-10% of all intracranial aneurysms. The most common sites where these lesions occur are the bifurcation of the basilar artery and the origin of the PICA. When present in the distal portion of the PICA, they have been reported to represent from 0.5% to 6%. These lesions are referred to as giant when they exceed 25 mm in diameter. Most of these aneurysms present as partially thrombosed, but sometimes they can be occluded, making differential diagnosis with tumors a defying task [4].

Giant intracranial aneurysms often present with brainstem mass effect symptoms, multiple cranial nerve palsies, brainstem dysfunction, or hydrocephalus, which can be mistaken for neoplasms, as illustrated in our case, especially those that are entirely thrombosed [5,6]. Only less than 10% present with symptoms of subarachnoid hemorrhage [7]. Haley et al. reported a case of central sleep apnea secondary to compression of respiratory centers in the medulla [8]. Gambhir et al. reported a case of intractable hiccups due to irritation of the vagal nerve nuclei [9]. The cases mentioned above demonstrate the wide range of symptoms observed in PICA giant aneurysms.

Murrone et al. reported in their study that only 17 cases, in addition to the one previously published by them, of distal PICA aneurysms treated surgically have been reported so far. In most cases, good results are achieved, demonstrating that despite being a rare pathology, surgical management in highly specialized centers with vascular neurosurgeons is a viable option [4].

Recent studies proposed using methods such as standard endosaccular occlusion or stenting to manage vertebrobasilar circulation aneurysms by endovascular therapy. Endovascular parent artery sacrifice is a good option for distal PICA aneurysms, as the length and tortuosity of the vessel make selective catheterization unsuitable. If an occlusion occurs, it is typically well-tolerated clinically. On the other side, surgical management remains the mainstay of treatment when compressive symptoms due to the mass effect are present, as in the present case. Hall et al. also mentioned that giant distal PICA aneurysms can be managed surgically with relatively low morbidity and good results [10].

## Conclusions

Giant aneurysms may be approached in many ways. CT scans appear as round- or oval-shaped masses, with distinct appearances depending on the amount of intra-aneurysmal thrombosis and can be easily mistaken for neoplastic lesions. Partially thrombosed aneurysms may enhance moderately and show the “target sign,” while complete thrombosis may appear as a homogeneous mass without enhancement. However, a CT scan may not be enough to make a specific diagnosis. Angiography remains the most accurate diagnostic modality to get to a precise diagnosis. It shows the origin and definitive characteristics of the lesion used in the treatment planning.
